# Molecular Epidemiology and Characteristics of CTX-M-55 Extended-Spectrum β-Lactamase-Producing *Escherichia coli* From Guangzhou, China

**DOI:** 10.3389/fmicb.2021.730012

**Published:** 2021-10-11

**Authors:** Shihan Zeng, Jiajun Luo, Xiankai Chen, LiShao Huang, Aiwu Wu, Chao Zhuo, Xiaoyan Li

**Affiliations:** ^1^KingMed School of Laboratory Medicine, Guangzhou Medical University, Guangzhou, China; ^2^Department of Clinical Laboratory, Fifth Affiliated Hospital, Southern Medical University, Guangzhou, China; ^3^State Key Laboratory of Respiratory Disease, First Affiliated Hospital of Guangzhou Medical University, Guangzhou, China

**Keywords:** CTX-M-55, IncI1 plasmid, IS*Ecp1*, ST1193, *E. coli*, IncFII plasmid

## Abstract

In recent years, the CTX-M-55 extended-spectrum β-lactamase (ESBL)-positive rate has gradually increased in the clinic. To identify the molecular epidemiology and characteristics of *bla*_CTX–M__–55_-positive isolates, a total of 374 non-repetitive ESBL-producing *Escherichia coli* strains were collected from patients in two hospitals in Guangzhou, and 89 *bla*_CTX–M__–55_-positive isolates were selected by CTX-M-1-group PCR amplification and confirmed by DNA sequencing. Whole-genome sequencing was used to analyze the resistance phenotype, plasmid types, phylogenetic relationships and genetic environment of the *bla*_CTX–M__–55_ gene. Conjugation experiments and PCR were performed to confirm whether the plasmid harboring *bla*_CTX–M–55_ gene could be transferred. The results showed that all *bla*_CTX–M–55_-positive isolates were resistant to ceftriaxone, and 88.76 and 76.40% were resistant to ceftazidime and cefepime, respectively. The resistance rates to levofloxacin and sulfamethoxazole were 66.29 and 59.55%, respectively. However, the sensitivity rate of piperacillin/tazobactam, amoxicillin/clavulanate, and amikacin exceeded 90%. All *bla*_CTX–M–55_-positive isolates were sensitive to carbapenems. Thirty-two STs were detected in the *bla*_CTX–M–55_-positive isolates, among which the detection rate of ST1193 was relatively high (19.10%, 17/89), and other ST types were scattered. It remains to be seen whether ST1193 carrying the *bla*_CTX–M__–55_ gene can become a popular clone strain in this region in the future. The plasmid types carrying the *bla*_CTX–M__–55_ gene included IncI1, IncFII, IncFIC, IncFIB, IncHI2, IncI2, and IncX/Y, among which the IncI1 and IncFII plasmids were the main plasmids, accounting for 37.80 and 28.09%, respectively. Among them, 11 strains of the IncI1 plasmid existed in ST1193 strains. The *bla*_CTX–M__–55_ gene was found on chromosomes of 13 isolates, and seemed to be increasing annually. Up to five distinct types of genetic environments surrounding the *bla*_CTX–M__–55_ gene were analyzed. The most common structure was type II “IS*Ecp1*-*bla*_CTX–M__–55_-ORF477.” In conclusion, whether ST1193, which carries *bla*_CTX–M__–55_ gene, will be an epidemic clone of this region in the future remains to be concerned. The plasmids IncI1 and IncFII, and mobile elements such as IS*Ecp1* and IS*26* may be the main factors leading to the spread and prevalence of CTX-M-55 genotypes.

## Introduction

Extended-spectrum β-lactamases (ESBLs), including TEM, SHV, CTX-M, and OXA enzymes, are the main resistance mechanism of Enterobacteriaceae against β-lactam antibiotics. Among them, CTX-M type β-lactamase, which was first found in 1990 by Bauernfeind et al. (1990), could preferentially hydrolyze cefotaxime (CTX) compared with TEM- and SHV-type enzymes. The CTX-M type has been reported to be the main type of ESBL in Enterobacteriaceae spreading worldwide and is also widely distributed in zoonotic pathogens (Bernard et al., 1992; Rossolini et al., 2008). CTX-M-14 and CTX-M-15 have been reported to be the most common genotypes in China in recent years. However, the CTX-M-55 positive rate has gradually increased in China, especially in the area of South China (Zhang et al., 2014). In Zhang et al. (2014), Cao et al. (2011), and Zhao and Hu (2013), the detected positive rate of CTX-M-55 was even higher than that of its derivative type, CTX-M-15. CTX-M-55 is a variant of CTX-M-15 with only one amino acid substitution (Ala-80-Val). Both CTX-M-15 and CTX-M-55 belong to the CTX-M-1 group, but CTX-M-55 exhibits high hydrolytic activity to ceftazidime (Kiratisin et al., 2007). Since CTX-M-55 was first reported in Thailand in 2006, it has been identified in *Escherichia coli*, *Klebsiella pneumoniae*, *Salmonella*, and *Morganella morganii* (Kiratisin et al., 2007; Kim J. S. et al., 2017; Xia et al., 2017; Hu et al., 2018). Plasmids are known as an important reason for the rapid spread of *bla*_CTX–M_. Recently, CTX-M-55 has been reported to appear on the IncI1, IncF, IncP, and IncA/C plasmids (Kim J. S. et al., 2017; Xia et al., 2017; Hounmanou et al., 2021). Additionally, the transmission of *bla*_CTX–M_ is also related to many mobile genetic elements. For example, IS*Ecp1*, IS*26*, and IS*903* are often detected around *bla*_CTX–M_ (Lartigue et al., 2006; Poirel et al., 2008; Hu et al., 2018). In this study, 89 *bla*_CTX–M__–55_-producing *E. coli* isolates isolated from patients in two hospitals in Guangzhou in recent years were selected, and the epidemiology and characteristics of these isolates were analyzed.

## Materials and Methods

### Bacterial Strains and Antimicrobial Susceptibility Testing

A total of 374 non-repetitive ESBLs-producing *E. coli* strains isolated from patients in two hospitals in Guangzhou were identified by the advanced expert system (AES) of the VITEK-2 COMPACT Automatic Microbial Identification System (bioMérieux, Marcy-l’Étoile, France). Minimal inhibitory concentrations (MICs) including the ESBLs-resistant phenotype were determined using the VITEK-2 Automated Susceptibility System (Spanu et al., 2006). The MICs of the isolates against amoxicillin/clavulanic acid (AMC), piperacillin/tazobactam (TZP), amikacin (AMK), compound sulfamethoxazole (SXT), ceftriaxone (CRO), ceftazidime (CAZ), cefepime (FEP), ertapenem (ETP), imipenem (IPM), and levofloxacin (LVX) were determined by agar dilution method, and the results were interpreted according to the Clinical and Laboratory Standards Institute (CLSI/NCCLS M100-S30) [Clinical and Laboratory Standards Institute [CLSI], 2021]. All isolates were collected in two tertiary hospitals from 2012 to 2017 and 2020, while the isolates from 2018 to 2019 were not collected, so they were not included in this study. *E. coli* ATCC 25922 was used as a quality control strain. *E. coli* C600 was used as the recipient strain in the conjugation experiments.

### Detection of the CTX-M-1-Group β-Lactamase Gene by PCR Assays

Primers for detecting all CTX-M-1-group type genes were designed are shown in Supplementary Table 1. Bacterial genomic DNA was extracted for PCR amplification and the positive PCR products were subsequently sequenced and to confirm their phenotype. Only *bla*_CTX–M__–55_-positive isolates were selected for subsequent testing.

### Conjugation Experiments

The transferability of the *bla*_CTX–M__–55_ gene was determined by conjugation experiments with rifampin-resistant *E. coli* C600 as the recipient strain. Transconjugants were selected on Luria–Bertani agar plates containing rifampin (100 μg/mL) and CRO (4 μg/mL). PCR using CTX-M-1-group primers and sequencing were used to confirm whether the transconjugants carrying the *bla*_CTX–M__–55_ gene were successfully acquired. Antimicrobial susceptibility testing and plasmid replicon typing (primers are in Supplementary Table 1) were conducted on transconjugants. The presence of resistant genes from transconjugants was also investigated by PCR (primers showed in Supplementary Table 1).

### Whole-Genome Sequencing and Analysis

The genomic DNA of *bla*_CTX–M__–55_-positive *E. coli* was extracted by a bacterial genomic DNA extraction kit (Tiangen, Beijing, China) and sequenced by next-generation sequencing (NGS) on an Illumine platform (Nuohezhiyuan, Tianjin, China). The quality of the raw readings was controlled by the interactive program FastQC (Wingett and Andrews, 2018), and the genomes were assembled using SPAdes 3.13.1 (Bankevich et al., 2012) and annotated using Prokka 1.14.5 (Seemann, 2014) on the Lunix system. Multilocus sequence typing analysis of the *E. coli* isolates was executed using MLST 2.18.0 (Larsen et al., 2012). The core genome multilocus sequence typing (cgMLST) of 17 ST1193 isolates was performed using Ridom SeqSphere+ 4.1.9 (Junemann et al., 2013). The resistance genes and plasmids type were determined based on the CGE server (Thomsen et al., 2016), and the plasmid circle map illustrates with BRIG (Alikhan et al., 2011). In all second generation genome annotation files, contigs harboring the *bla*_CTX–M__–55_ gene were analyzed, and the *bla*_CTX–M__–55_ gene locations were roughly determined combined with BLAST.^1^ Representative isolates with unclear *bla*_CTX–M__–55_ gene locations or with different *bla*_CTX–M__–55_-harboring plasmid types were selected and sequenced for long read sequencing on the Nanopore platform (Nuohezhiyuan, Tianjin, China). Finally, the location and genetic environment of the *bla*_CTX–M__–55_ gene in *bla*_CTX–M__–55_-positive *E. coli* were analyzed based on second- and third-generation genomic data. The genetic environment of the *bla*_CTX–M__–55_ genes was drawn by Easyfig (Sullivan et al., 2011).

## Results

### *bla*_CTX–M__–55_-Positive *Escherichia coli* Isolates

**FIGURE 1:**
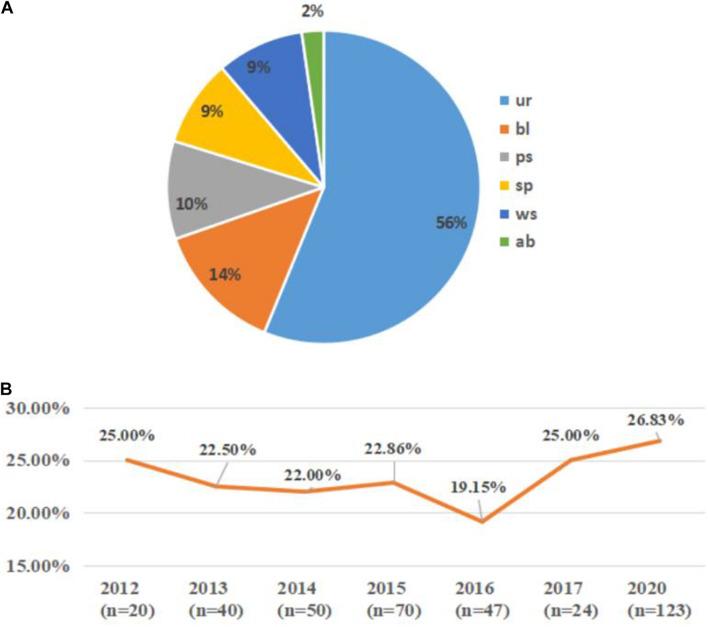
The distribution of specimen sources and collection years of the *bla*_CTX–M__–55_-positive isolates. **(A)** The specimen source distribution of all the *bla*_CTX–M__–55_-positive isolates. ur, urine; bl, blood; ps, purulent secretion; sp, sputum; ws, wound secretion; ab, abdominal drainage fluid. **(B)** The collection year’s distribution of the *bla*_CTX–M__–55_-positive isolates among all the isolates. *n* indicates the number of isolates collected in that year; orange line indicates the proportion of *bla*_CTX–M__–55_-positive isolates among the ESBL-producing *E. coli* isolates collected in that year (missing data from 2018 to 2019).

A total of 132 *bla*_CTX–M__–1–group_-positive ESBLs-producing *E. coli* (35.29%) isolates were obtained from all 374 ESBLs-producing *E. coli* clinical isolates. Overall, 6 *bla*_CTX–M__–3_-positive isolates (1.60%), 37 *bla*_CTX–M__–15_-positive isolates (9.89%), and 89 *bla*_CTX–M__–55_-positive isolates (23.80%) were identified. In this study, 89 *bla*_CTX–M__–55_-positive isolates were further analyzed, and the distribution of their specimen source and collection year are shown in Figure 1. The *bla*_CTX–M__–55_-positive isolates were derived mainly from patient urine (56%), while other sources included blood (14%), purulent secretion (10%), sputum (9%), wound secretion (9%), and abdominal drainage fluid (2%).

### MLST Profile

**FIGURE 2:**
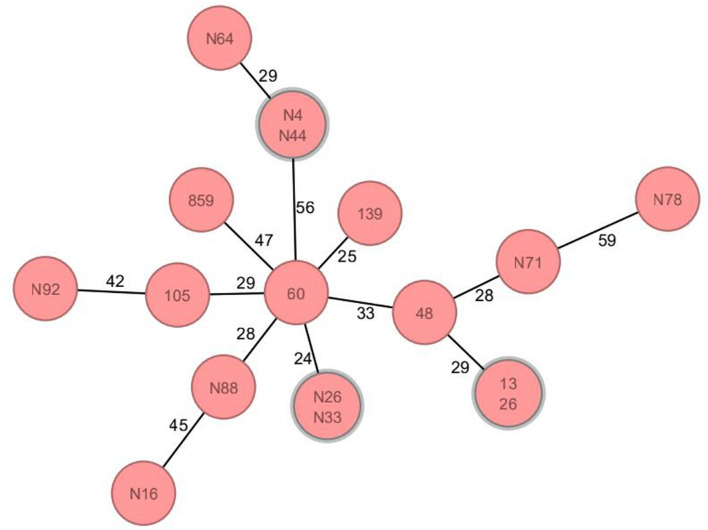
Core genome multilocus sequence typing analysis of ST1193 isolates carrying *bla*_CTX–M__–55_ gene. *Circles* represent ST1193 isolates, and the number on each *circle* connecting *line* is the allele difference between the two isolates. Clonally related genetic complexes (=15 alleles difference) containing more than one patient are encircled in *gray*. There were no differential alleles between three pairs of isolates (N4&N44, N26&N33, and 13&26); furthermore, the number of differential alleles in other isolates was more than 15.

Thirty-two MLST profiles were determined from the 89 *bla*_CTX–M__–55_-positive isolates (Supplementary Table 2), including three novel STs (ST12284, ST12285, and ST12303), of which sequences have been submitted to the PubMLST database.^2^ In addition to the relatively high detection rate of ST1193 (19.10%, 17/89), other ST types were scattered. It is worth noting that ST1193 isolates mainly emerged in 2020 (10/17) and were distributed in different years (Supplementary Table 2). By cgMLST, among 17 ST1193, except for 3 pairs of strains that showed the same alleles, the rest ST1193 strains isolated from different years still had different alleles (Figure 2).

### Antimicrobial Susceptibility Profiles

**FIGURE 3:**
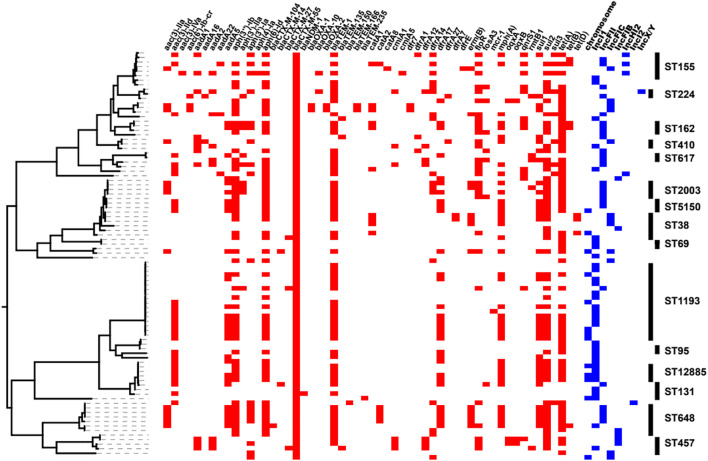
Heatmaps of partial resistance genes, ST types and *bla*_CTX–M__–55_ gene locations combined with the phylogenetic tree of the *bla*_CTX–M__–55_ isolates. The phylogenetic tree was drawn using PARSNP software. The *red box* indicates that the isolate contains relevant resistance gene, and the *blue box* indicates the location of the *bla*_CTX–M__–55_ gene in the isolate. The rightmost end of the figure shows the ST type (*n* ≥ 2) of the isolates.

All of the *bla*_CTX–M__–55_-positive isolates were resistant to CRO, and 88.76 and 76.40% were resistant to CAZ and FEP, respectively (Supplementary Table 3). The resistance rates to LVX and SXT were 66.29 and 59.55%, respectively. However, the *bla*_CTX–M__–55_-positive isolates presented high susceptibility rates to AMC, TZP, and AMK, with sensitivity rates of 95.95, 95.40, and 94.38%, respectively. All *bla*_CTX–M__–55_-positive isolates were sensitive to IPM and ETP. Meanwhile, we found that *bla*_TEM_ (coding β-lactamase genes), *sul*, and *dfrA* (mediating sulfonamide resistance), *tet* (mediating tetracycline resistance), *aph(3″)-Ib* and *aph(6′)-Ib* (mediating aminoglycoside resistance) were abundant in *bla*_CTX–M__–55_-positive isolates (Figure 3 and Supplementary Table 3). The 16S rRNA methylase gene *rmtB* (mediating aminoglycoside antibiotic resistance) was detected in five isolates, all of which were resistant to AMK (Supplementary Table 3). There were sixteen isolates containing genes mediating fluoroquinolone resistance, of which three were *aac(6′)-Ib-cr* and thirteen were *qnrS* (Figure 3 and Supplementary Table 3). *dfrA* or *sul* genes were detected in almost all SXT resistant isolates (52/53), and 46 isolates were detected in both of them.

### The *bla*_CTX–M__–55_ Gene Location

**FIGURE 4:**
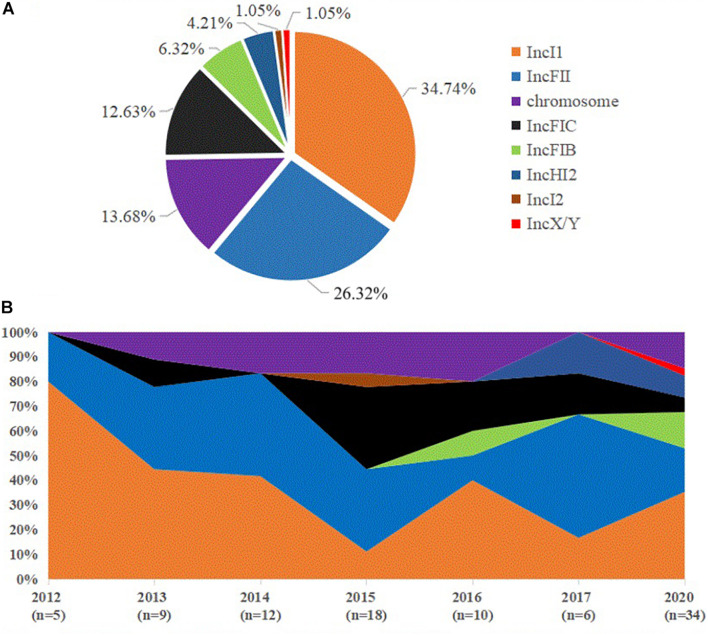
Distribution of *bla*_CTX–M–55_ gene location and dynamic changes in the incompatible group of plasmids in all the *bla*_CTX–M–55_-positive *E. coli* isolates. **(A)** The percentage of *bla*_CTX–M–55_ gene location found in all *bla*_CTX–M__–55_-positive *E. coli* isolates. **(B)** Percentage dynamics in major incompatible plasmids and chromosomes per year (missing data from 2018 to 2019).

The predicted plasmid components carrying the *bla*_CTX–M__–55_ gene were of variable replicon types including IncI1, IncFIB, IncFII, IncFIC, IncHI2, IncI2, and IncX/Y (Figure 4A). The *bla*_CTX–M__–55_ gene of 33 isolates was located on the IncI1 plasmid, among which 11 were carried by ST1193. The other *bla*_CTX–M__–55_-positive plasmid was identified, 24 on the IncFII plasmid, 12 on the IncFIC plasmid, 6 on the IncFIB plasmid, 3 on the IncHI2 plasmid, 1 on the IncI2 or IncX/Y plasmid, and 1 simultaneously on the IncHI2 and IncFII plasmids (Supplementary Table 2). These plasmids were randomly present in different ST isolates. We also found that in thirteen of the 89 isolates containing the *bla*_CTX–M__–55_ gene, BLSTA in NCBI suggested a chromosomal location (Figure 3 and Supplementary Table 2). Among them, the *bla*_CTX–M__–55_ gene of three isolates was simultaneously located on both the chromosome and the IncI1 plasmid, and two were simultaneously located on the chromosome and the IncFIC plasmid. The isolates with the chromosome-encoding *bla*_CTX–M__–55_ gene seemed to increase annually (Figure 4B). Our data (Figure 4B) showed that the total diversity of the plasmid types carrying the *bla*_CTX–M__–55_ gene gradually increased between 2012 and 2020 (missing data from 2018 to 2019). Especially, the IncI1 and IncFII plasmids were continuously identified with higher detection rates. The IncFIB and IncHI2 emerged in the last 5 years, and the detection rate for these plasmids was still relatively low.

**FIGURE 5:**
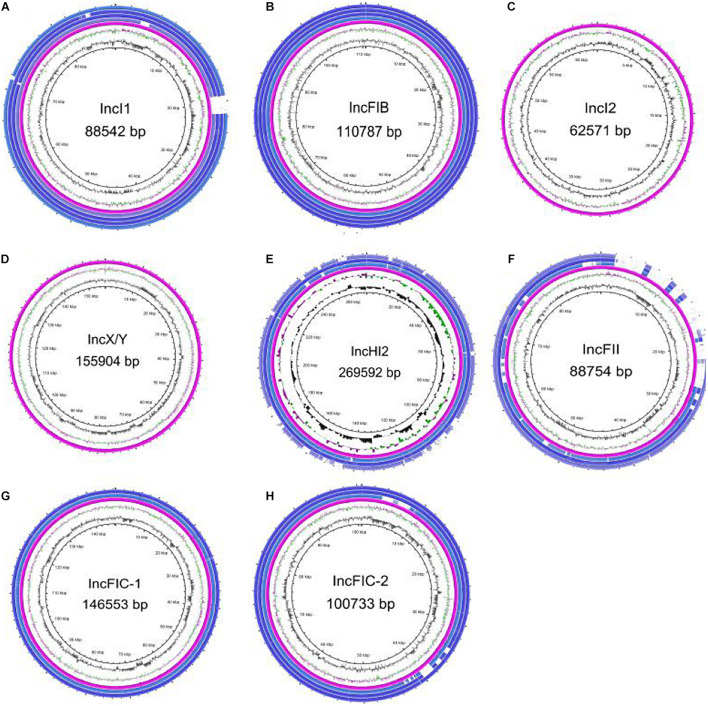
Plasmid backbone comparison between different *bla*_CTX–M–55_-carrying plasmids. All types of plasmid circles were based on the known full-sequence plasmids as reference sequences from this study, and several isolates with the same type of plasmid were selected for drawing through BRIG. Among them, the IncI2 and IncX/Y plasmids contained only reference plasmids, and the IncFIC plasmid had two backbone types. **(A)** IncI1 plasmid. **(B)** IncFIB plasmid. **(C)** IncI2 plasmid. **(D)** IncX/Y plasmid. **(E)** IncHI2 plasmid. **(F)** IncFII plasmid. **(G)** IncFIC plasmid, the first backbone. **(H)** IncFIC plasmid, the second backbone.

Circle diagrams were drawn for plasmid backbone comparison based on the known full-sequence plasmids in this study as reference. The IncI1 (Figure 5A), IncFIB (Figure 5B), IncI2 (Figure 5C), IncX/Y (Figure 5D), and IncHI2 (Figure 5E) plasmids had only one backbone type, while the IncFIC plasmid had two backbone types (Figures 5G,H). Most plasmids carrying *bla*_CTX–M__–55_ with the same replicon were relatively conserved in this study, such as IncI1, IncFIB and IncHI2. In particular, almost all of the IncI1 plasmids were highly similar to p2474-3 (CP021208) in clinical *E. coli* isolated from a hospital in Anhui Province. Incidentally, we found that the sequence similarity between the IncI1 plasmid p628-CTXM (KP987217) isolated from *K. pneumoniae*, pST53-2 (CP050747.1) isolated from *Salmonella enteritidis* and p2474-3 was higher than 95%, and there was only one resistance gene *bla*_CTX–M__–55_ between them. In contrast, the backbones of the four IncFII plasmids (Figure 5F) were slightly different, with a distinct region of approximately 24 kbp containing 24 coding sequences (CDSs). This region included some mobile genes (IS*26*, *ISEc36*, IS*Kpn19*, IS*6100*, IS*SBbo1*, IS*50R*, and Tn2), the *neo* gene encoding aminoglycoside 3′-phosphotransferase, the *hin* gene encoding a specific recombinase, the *hacb* gene encoding a dehydrogenase, the *tet(M)* gene mediating tetracycline resistance, the *tap* gene encoding a multidrug efflux pump, and eight hypothetical protein. Meanwhile, we found that the IncFII plasmid carrying the *bla*_CTX–M__–55_ gene also contains other drug resistance genes, such as *bla*_TEM__–1_, *fos(A)*, *qnrS1*, *tet*, *dfrA*, or *catA* genes by analyzing the long read sequence (Supplementary Tables 2, 3).

### Transconjugants of the *bla*_CTX–M__–55_-Positive Isolates

In total, 45 transconjugants were successfully obtained from 89 *bla*_CTX–M__–55_-positive isolates through conjugation experiments, and the transfer success rate was 50.56% (45/89). Combined with the *bla*_CTX–M__–55_ gene location and the plasmid replicon typing results of transconjugants (Supplementary Table 2), it was found that most of the IncI1 (24/33, 72.72%) and IncFII plasmids (16/25, 64%) could be transferred into *E. coli* C600, while the transfer success rate of the IncFIC and IncFIB plasmids was lower than 33.33%. In this study, the IncHI2 (0/4), IncI2 (0/1), and IncX/Y (0/1) plasmids carrying the *bla*_CTX–M__–55_ gene failed to transfer through conjugation experiments. Meanwhile, there is not only one plasmid in some transconjugants. All transconjugants were resistant to CRO. The transconjugants exhibited 95.56 and 82.22% resistance rates to CAZ and FEP, respectively. Only 4.44% of the transconjugants were resistant to AMC. In addition, the resistance rates of transconjugants to SXT, LEV, and AMK were 17.78, 6.67, and 2.22%, respectively. All transconjugants were sensitive to TZP, IPM, and ETP (Supplementary Table 3).

A total of 10 transconjugants were resistant to at least one antibiotic in LEV or SXT. Interestingly, the *bla*_CTX–M__–55_ gene of seven transconjugants was located on IncFII, which means that in addition to the *bla*_CTX–M__–55_ gene, IncFII in these transconjugants also carries genes that mediate LVX or SXT resistance (Supplementary Tables 2, 3). Analysis of drug resistance genes in transconjugants showed that the *dfrA17*/*dfrA14* gene are the main reason for the resistance of transconjugants to SXT. There were three transconjugants resistant to LEV, and *qnrS1* gene was detected in all of them. Only one transconjugant carried the *rmtB* gene and was resistant to amikacin.

### Genetic Environment Surrounding the *bla*_CTX–M__–55_ Gene

**FIGURE 6:**
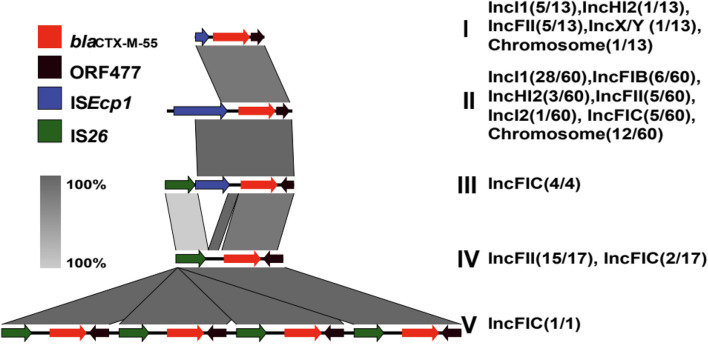
Genetic environment surrounding the *bla*_CTX–M__–55_ gene. The *colored arrow* represents the ORFs and the arrow direction indicates the transcription direction. A total of 6 *bla*_CTX–M__–55_ genetic structures were obtained, and different genetic environments surrounding the *bla*_CTX__–M__–55_ gene exist in different types of plasmids. The architecture of type I (13/95) is “ΔIS*Ecp1*-*bla*_CTX–M__–55_-ORF477”; type II (60/95) is “IS*Ecp1*-*bla*_CTX–M__–55_-ORF477”; type III (4/95) is “IS*26*-ΔIS*Ecp1*-*bla*_CTX–M__–55_-ORF477”; type IV (17/95) is “IS*26*-*bla*_CTX–M__–55_-ORF477”; type V (1/95) is “IS*26*-*bla*_CTX–M–55_-ORF477-IS*26*-*bla*_CTX–M–55_-ORF477- IS*26*-*bla*_CTX–M–55_-ORF477- IS*26*-*bla*_CTX–M–55_-ORF477.”

The genetic environment surrounding the *bla*_CTX–M–55_ gene is presented in Figure 6. Five structures were obtained by analyzing mobile elements around the *bla*_CTX–M–55_ gene and named type I to V. The mobile elements located upstream of *bla*_CTX–M–55_ mainly included IS*Ecp1* (complete or incomplete) and IS*26*. Downstream of the *bla*_CTX–M–55_ genes ORF477 was consistently found. Among them, type II “IS*Ecp1*-*bla*_CTX–M–55_-ORF477” was the predominant (63.16%, 60/95) genetic environment of the *bla*_CTX–M–55_ gene and plasmids containing this structure included IncI1, IncFIB, IncFIC, IncFII, IncHI2, and IncI2 (Figure 6). Likewise, the genetic environment of the *bla*_CTX–M–55_ gene on the chromosome (12/13) was almost type II, the other is type I. Compared with type II, only a large deletion (489 to 1140 bp) of IS*Ecp1* was found in type I. Moreover, the *bla*_CTX–M–55_ genes of isolate 75, 128, and 173 were found on both the chromosome and the IncI1 plasmid, and both of the genetic environments between them belong to type II. The *bla*_CTX–M–55_ gene of isolate N18 was found on both the chromosome and the IncFIC plasmid, among which the genetic environment on the chromosome was type II, and that on the IncFIC plasmid was type III “IS*26*-ΔIS*Ecp1*-*bla*_CTX–M–55_-ORF477.” The occurrence of the type III structure was similar to that of the type II structure, but IS*Ecp1* of the type III structure was disrupted by IS*26*. Interestingly, IS*26* mainly emerged upstream of the *bla*_CTX–M–55_ gene in the IncFIC and IncFII plasmids. Type IV “IS*26*-*bla*_CTX–M–55_-ORF477” mainly exists in IncFII plasmids (15/17).

Notably, an IncFIC plasmid contained four copies of the *bla*_CTX–M__–55_ gene (Type V) harbored in isolate 110, and the structure of the four-duplicated segment was “IS*26*-*bla*_CTX–M__–55_-ORF477.” The plasmid backbone was similar to that of IncFIC-2 (Figure 5H) and 151563 bp in size. This plasmid cannot be transferred through conjugation. The MIC of isolate 110 for CRO was higher than 256 μg/mL, and the MICs for CAZ and FEP were 64 μg/mL. Compared to the common *bla*_CTX–M__–55_-positive isolates with CRO ≥ 64 μg/mL, and the MIC_50_ for CAZ was 32 μg/mL, and the MIC_50_ for FEP was only 16 μg/mL.

## Discussion

Previous studies (Lartigue et al., 2004; Bevan et al., 2017) have shown that the isolation rate of Enterobacteriaceae producing CTX-M-55 in China has increased significantly in recent years, in aquaculture animals and in clinical patients, which demonstrates the rapid dissemination of *bla*_CTX–M__–55_, especially in South China (Zhang et al., 2014). In that study (Zhang et al., 2014), the CTX-M-55 (21/38, 55.26%) positive rate in clinical ESBLs-producing isolates from Guangdong Province (in South China) was much higher than that from other provinces in China (range from 2.56 to 31.25%). Previous study (Zhang et al., 2014) have shown that the percentage of *bla*_CTX–M__–55_-positive *E. coli* reported in China was 18.40% (47/256), exceeding the percentage of *bla*_CTX–M__–__1__5_-positive *E. coli* (31/256, 12.1%), which has been reported as the most widespread CTX-M gene in *E. coli* in China. In this study, we obtained similar results with a more significant trend: the detection rate of *bla*_CTX–M__–55_-positive isolates reached 23.80% (89/374), which was higher than that of *bla*_CTX–M__–__1__5_-positive isolates (37/374, 9.89%).

Most *bla*_CTX–M__–55_-positive isolates have been reported co-harboring *bla*_TEM_ (Hu et al., 2018), and similar results were shown in this study. Except for *bla*_TEM_, *aph (3′)-Ib*, *aph(6′)-Ib*, *dfrA*, *sul*, and *tet* mediating different antibiotic resistances emerged frequently in the *bla*_CTX–M__–55_-positive isolates. All of the *bla*_CTX–M__–55_-positive isolates were resistant to CRO, and 88.76 and 76.40% were resistant to CAZ and FEP, respectively. The production of ESBLs is the main mechanism of resistance against cephalosporin in *E. coli*, and CTX-M-55 producing *E. coli* are known to be resistant to the second and third generations of cephalosporin (Wang et al., 2013). Cefepime is the first fourth-generation cephalosporin approved for use in China, showing low toxicity and high activity against third-generation cephalosporin-resistant Enterobacteriaceae (Chapman and Perry, 2003). However, the resistance rates of *bla*_CTX–M__–55_-positive isolates to FEP reached 76.40% in this study, which showed that the resistance phenomenon of *bla*_CTX–M__–55_-positive isolates to cephalosporins became tougher. In addition, *bla*_CTX–M__–55_-positive isolates often carry *sul* and *dfrA* genes, which mediate their resistance to SXT up to 66.09%. Even though there are few *bla*_CTX–M__–55_-positive isolates carrying *qnrS* or *aac(6′)-Ib-cr* gene, the resistance to fluoroquinolone reached 59.55%. Because fluoroquinolones are mainly mediated by plasmid mediated drug resistance genes (PMQRs) and mutations in quinolone resistance determining regions (QRDRs), and PMQR only confers low-level fluoroquinolone resistance (Robicsek et al., 2006; Strahilevitz et al., 2009; Zhang et al., 2019). Fortunately, the sensitivity of *bla*_CTX–M__–55_-positive isolates to AMC, TZP, and AMK was higher than 90%, and all *bla*_CTX–M__–55_-positive isolates were sensitive to IPM and ETP. Therefore, β-lactamase inhibitors, amikacin, and carbapenems can be used as a treatment strategy for *bla*_CTX–M__–55_-positive isolates. The results of resistance analysis indicate that the resistance genes carried by *bla*_CTX–M__–55_-positive clinical isolates are diverse and complex, which will bring great challenges to clinical treatment.

Previous studies (Russo and Johnson, 2003) showed that the population structure of CTX-M-producing *E. coli* is dominated by the high-risk clone ST131, and other important epidemic clones included ST405, ST38, ST648, ST410, and ST1193 (Coque et al., 2008). However, the detection rate of ST131 in this study was only 4.94% (4/89). ST1193 (19.10%) was detected much higher than that of other strains. ST1193 was reported as a fluoroquinolone-resistant *E. coli* clone (Platell et al., 2012; Kim Y. et al., 2017; Tchesnokova et al., 2019) and it is commonly coresistant to sulfonamides, β-lactams, and tetracyclines (Tchesnokova et al., 2019). The *bla*_CTX–M__–55_-positive *E. coli* isolates carried by patients with urinary tract infections was reported in China (Xia et al., 2017), ST1193 (18%) was also the most common ST. ST1193 is often associated with clinical isolates of urinary tract infections (Ding et al., 2021). The *bla*_CTX–M__–55_-positive isolates were obtained from multiple clinical specimens in this study. Of the 17 ST1193 isolates, 10 were derived from urine specimens. The results of cgMLST analysis showed that, except for the three pairs of strains, the other *bla*_CTX–M__–55_-positive ST1193 clones isolated from different time and departments exist allelic difference, which indicate that although the genetic relationship of these ST1193 strains were closely related, they still had different degrees of mutation over time. We also found that a total of 10 isolates of ST1193 were detected in 2020 and 7 isolates in 2012–2017 (Supplementary Table 2). Therefore, we speculate that ST1193 may become a potential epidemic clone among clinical isolates carrying *bla*_CTX–M__–55_ in the future, but it still needs further observation. Furthermore, this study showed that the *bla*_CTX–M__–55_ gene is located on the chromosome with an increasing trend, and a study of *bla*_CTX–M__–55_ from *Salmonella* (Zhang et al., 2019) also showed that *bla*_CTX–M__–55_ mainly existed in the chromosome (10/11), which suggests that we should pay close attention to the vertical transmission of *bla*_CTX–M__–55_-positive strains.

Conjugative plasmids play a key role in the horizontal transfer of drug resistance genes among *E. coli* (Carattoli, 2013), which is an important reason for *bla*_CTX–M_ transmission. In recent years, *bla*_CTX–M__–55_ in China has mainly been located on the IncI1 plasmid and sporadically emerged on the IncF and IncP plasmids (Bevan et al., 2017; Xia et al., 2017). The plasmid types carrying the *bla*_CTX–M__–55_ gene found in this study included IncI1, IncFII, IncFIC, IncFIB, IncHI2, IncI2, and IncX/Y. Among them, the detection rates of IncI1 and IncFII plasmids were the highest. Meanwhile, most IncI1 and IncFII plasmids can be transferred to *E. coli* C600, but the transfer success rate of other plasmids was low. We also found plasmids similar to IncI1 in *K. pneumoniae* and *S. enteritidis* (Fu et al., 2020), and the sequence identity was higher than 95%, indicating that the IncI1 plasmid carrying *bla*_CTX–M__–55_ can be spread in different strains. The IncI1 plasmid carrying only one drug resistance gene of *bla*_CTX–M–55_ in Enterobacteriaceae is still relatively conserved after several years of transmission. In this study, 64.71% (11/17) of ST1193 contained IncI1 plasmids, while 88% (14/16) of the ST1193 isolates studied by Xia et al. (2017) also contained IncI1 plasmids. In addition, many IncI1 plasmids (22/33, 66.67%) were distributed in other ST types. Our results suggest that the dominant plasmid IncI1 may be one of the main reasons for the widespread dissemination of the *bla*_CTX–M__–55_ gene in *E. coli*. Additionally, the IncFII plasmid can be spread in many ST isolates. Meanwhile, it should be noted that the IncFII plasmid carrying the *bla*_CTX–M__–55_ gene also contains other drug resistance genes, such as *bla*_TEM__–1_, *fos(A)*, *qnrS1*, *tet*, *dfrA*, or *catA* genes by analyzing the long read sequence (Supplementary Tables 2, 3). Likewise, the analysis of the results of transconjugants showed that the coexistence of the *bla*_CTX–M__–55_ gene and other resistance genes (*dfrA17*, *dfrA14*, *qnrS1*, *tet*, and *rmtB*) seemed to be the most common on the IncFII plasmid. Therefore, isolates containing the IncFII plasmid carrying *bla*_CTX–M__–55_ were more resistant to clinical antibiotics, and it should also be considered in the clinic.

Mobile sequences such as insertion sequences (ISs) and transposons (Tns) are important elements that mediate the horizontal transmission of the *bla*_CTX–M_ gene (Cullik et al., 2010; Ma et al., 2011). IS*Ecp1* is often located upstream of the CTX-M gene and is responsible for the movement of all *bla*_CTX–M_ genotypes (Zhao and Hu, 2013). At present, there are few studies exploring the genetic structure of the *bla*_CTX–M__–55_ gene in *E. coli*. Five types of genetic structure were obtained by analyzing the environment surrounding *bla*_CTX–M__–55_ in the isolates. The upstream elements were mainly IS*Ecp1* and IS*26*. Similar to previous studies (Cao et al., 2011; Fu et al., 2020), the most common genetic environment for *bla*_CTX–M__–55_ is type II “IS*Ecp1*-*bla*_CTX–M__–55_-ORF477.” Studies (Wang et al., 2013) have shown that IS*Ecp1* seems to be a strong activator of *bla*_CTX–M__–55_ expression. Additionally, IS*26*, IS*903*, and ORF477 are also detected frequently around them (Lartigue et al., 2006; Poirel et al., 2008; Hu et al., 2018). In this study, we found that the *bla*_CTX–M__–55_ gene existed on both chromosomes and plasmids of the same isolate. Interestingly, the genetic environments of isolates 75, 128, and 173 on the chromosome and on the IncI1 plasmid were the same, both of which were type II. When comparing the genetic environments of the chromosome and IncFIC plasmid of isolate N18, only one copy of IS*Ecp1* on the plasmid was disrupted by IS*26*. It has also been reported (Ensor et al., 2006; Zhang et al., 2019) that the disruption of the IS*Ecp1* element by IS*26* is related to the transmission of the *bla*_CTX__–M_ gene. IS*Ecp1* can mobilize the CTX-M gene to different types of plasmids with IS*26* (Canton et al., 2012). This result showed that under certain conditions, the mobile element may be able to mediate the transfer of the *bla*_CTX–M__–55_ gene between the plasmid and the chromosome. Although IS*903* was not detected, we found that IS*Ecp1* and IS*26* were upstream of the *bla*_CTX–M__–55_ gene. More interestingly, there were four copies of “IS*26*-*bla*_CTX–M__–55_-ORF477” in isolate 110. Generally, the presence of multi-copy *bla*_CTX–M__–55_ genes may be associated with increased cephalosporins resistance. The transposition mechanism of IS*26* is generally regarded to involve replicative transposition and co integrate formation (Jiang et al., 2020). We can speculate that IS*26* mediated transposon unequal crossover, produced four copies of IS*26* composite transposons in a row, which has also been reported in previous studies (Lee et al., 2012; Jiang et al., 2020). Therefore, we should strengthen the detection and analysis of mobile genetic element IS*26* to monitor the transmission trend of plasmids carrying the *bla*_CTX–M__–55_ gene with related elements in the clinical environment.

## Conclusion

Most of the isolates carrying the *bla*_CTX–M__–55_ gene are highly resistant to cephalosporins, but are still highly sensitive to amikacin, β-lactamase inhibitors and carbapenems, which can be used as the choice of clinical medication. In this study, prevalent ST clones were not detected. However, it remains to be seen whether ST1193 carrying the *bla*_CTX–M__–55_ gene can become a popular clone in this region. Meanwhile, we should pay close attention to the vertical transmission of *bla*_CTX–M__–55_-positive isolates. The epidemic plasmid IncI1 and IncFII, which have the highest detection rate and transfer efficiency, may play an important role in the spread of the *bla*_CTX–M__–55_ gene. In particular, the IncFII plasmid usually carries more drug resistance genes. Mobile elements such as IS*Ecp1* and IS*26* may be the main factors leading to the spread and prevalence of CTX-M-55 genotypes on chromosomes and plasmids.

## Data Availability Statement

The datasets presented in this study can be found in online repositories. The names of the repository/repositories and accession number(s) can be found below: https://www.ncbi.nlm.nih.gov/, JAHSRN000000000-JAHSSE000000000.

## Author Contributions

XL conceived and designed the study. CZ and AW provided the samples. XL and CZ funded the project. SZ and LH carried out the experiment. SZ, XC, and JL steered the literature search, data collection, and analysis. SZ drafted the manuscript. AW, XL, and CZ reviewed and approved the submission of the manuscript. All authors discussed the results and commented on the manuscript.

## Conflict of Interest

The authors declare that the research was conducted in the absence of any commercial or financial relationships that could be construed as a potential conflict of interest.

## Publisher’s Note

All claims expressed in this article are solely those of the authors and do not necessarily represent those of their affiliated organizations, or those of the publisher, the editors and the reviewers. Any product that may be evaluated in this article, or claim that may be made by its manufacturer, is not guaranteed or endorsed by the publisher.

## Footnotes

1
https://blast.ncbi.nlm.nih.gov/Blast.cgi

2
http://enterobase.warwick.ac.uk
