# Corrigendum: Molecular Epidemiology and Characteristics of CTX-M-55 Extended-Spectrum β-Lactamase-Producing *Escherichia coli* From Guangzhou, China

**DOI:** 10.3389/fmicb.2022.846287

**Published:** 2022-02-15

**Authors:** Shihan Zeng, Jiajun Luo, Xiankai Chen, LiShao Huang, Aiwu Wu, Chao Zhuo, Xiaoyan Li

**Affiliations:** ^1^KingMed School of Laboratory Medicine, Guangzhou Medical University, Guangzhou, China; ^2^Department of Clinical Laboratory, Fifth Affiliated Hospital, Southern Medical University, Guangzhou, China; ^3^State Key Laboratory of Respiratory Disease, First Affiliated Hospital of Guangzhou Medical University, Guangzhou, China

**Keywords:** CTX-M-55, IncI1 plasmid, IS*Ecp1*, ST1193, *E. coli*, IncFII plasmid

The author order was incorrectly listed as “Shihan Zeng, Jiajun Luo, Xiaoyan Li, Chao Zhuo, Aiwu Wu, Xiankai Chen and LiShao Huang”. The correct order is “Shihan Zeng, Jiajun Luo, Xiankai Chen, LiShao Huang, Aiwu Wu, Chao Zhuo, Xiaoyan Li^2^”. The author list and the correspondence section have been updated.

In the original article, there was an error. The sentence “There were only three single nucleotide differences between them.” is irrelevant.

A correction has been made to **Results, Genetic Environment Surrounding the bla**_**CTX**−**M**−**55**_ **Gene**, paragraph one:

“The genetic environment surrounding the *bla*_CTX−M−55_ gene is presented in Figure 6. Five structures were obtained by analyzing mobile elements around the *bla*_CTX−M−55_ gene and named type I to V. The mobile elements located upstream of *bla*_CTX−M−55_ mainly included IS*Ecp1* (complete or incomplete) and IS*26*. Downstream of the *bla*_CTX−M−55_ genes ORF477 was consistently found. Among them, type II “IS*Ecp1*-*bla*_CTX−M−55_-ORF477” was the predominant (63.16%, 60/95) genetic environment of the *bla*_CTX−M−55_ gene and plasmids containing this structure included IncI1, IncFIB, IncFIC, IncFII, IncHI2, and IncI2 (Figure 6). Likewise, the genetic environment of the *bla*_CTX−M−55_ gene on the chromosome (12/13) was almost type II, the other is type I. Compared with type II, only a large deletion (489 to 1140 bp) of IS*Ecp1* was found in type I. Moreover, the *bla*_CTX−M−55_ genes of isolate 75, 128, and 173 were found on both the chromosome and the IncI1 plasmid, and both of the genetic environments between them belong to type II. The *bla*_CTX−M−55_ gene of isolate N18 was found on both the chromosome and the IncFIC plasmid, among which the genetic environment on the chromosome was type II, and that on the IncFIC plasmid was type III “IS*26*-ΔIS*Ecp1*-*bla*_CTX−M−55_-ORF477.” The occurrence of the type III structure was similar to that of the type II structure, but IS*Ecp1* of the type III structure was disrupted by IS*26*. Interestingly, IS*26* mainly emerged upstream of the *bla*_CTX−M−55_ gene in the IncFIC and IncFII plasmids. Type IV “IS*26*-*bla*_CTX−M−55_-ORF477” mainly exists in IncFII plasmids (15/17).”

The authors apologize for this error and state that this does not change the scientific conclusions of the article in any way. The original article has been updated.

## Publisher's Note

All claims expressed in this article are solely those of the authors and do not necessarily represent those of their affiliated organizations, or those of the publisher, the editors and the reviewers. Any product that may be evaluated in this article, or claim that may be made by its manufacturer, is not guaranteed or endorsed by the publisher.

